# Unilateral Laminotomy with Bilateral Spinal Canal Decompression for Lumbar Stenosis: A Technical Note

**DOI:** 10.7759/cureus.623

**Published:** 2016-05-27

**Authors:** Marc Moisi, Christian Fisahn, Lara Tkachenko, R. Shane Tubbs, Daniel Ginat, Peter Grunert, Shiveindra Jeyamohan, Stephen Reintjes, Olaide Ajayi, Jeni Page, Rod J Oskouian, David Hanscom

**Affiliations:** 1 Seattle Science Foundation; 2 Neurological Surgery, Wayne State University; 3 Neurosurgery, Swedish Neuroscience Institute; 4 Department of Trauma Surgery, BG University Hospital Bergmannsheil, Bochum, Germany; 5 Neurosurgery, Seattle Science Foundation; 6 Radiology, University of Chicago; 7 Neurosurgery, Complex Spine, Swedish Neuroscience Institute

**Keywords:** lumbar stenosis, lumbar decompression, lumbar laminectomy, unilateral laminotomy bilateral decompression, lumbar spine, spinal stenosis

## Abstract

Lumbar stenosis has become one of the most common spinal pathologies and one that results in neurogenic claudication, back and leg pain, and disability. The standard procedure is still an open laminectomy, which involves wide muscle retraction and extensive removal of the posterior spinal structures. This can lead to instability and the need for additional spinal fusion. We present a systemized and detailed approach to unilateral laminotomy for bilateral decompression, which we believe is superior to the standard open laminectomy in terms of intraoperative visualization, postoperative stability, and degree of invasiveness.

## Introduction

Lumbar stenosis is one of the common spinal pathologies; it presents with back pain, leg pain, and neurogenic claudication [1-2]. Although different surgical modalities are available, the main objective of the operation is decompression of nerve roots and the spinal cord [3-4]. A surgical procedure that is linked with less morbidity related to postoperative deformity caused by disturbed spinal biomechanics has been advocated to preserve midline structures during a decompression [3]. Minimally invasive surgical procedures and microsurgical unilateral laminotomy with bilateral spinal canal decompression (ULBD) have been reported to achieve this goal [2, 4]. The objective of lumbar decompression is to decompress the neural elements while preserving stability and the spinous processes. It is our opinion that since L1-2, L2-3, and L3-4 are narrow, this is the procedure of choice. Bilateral laminotomies are indicated at L4-5 in selected cases if it is narrow. In this technical note, we report a modification of the procedure that we think improves visualization and therefore results in a better margin of safety. Informed consent was obtained from the patient for this study.

## Technical report

### Case illustration

**Figure 1:**
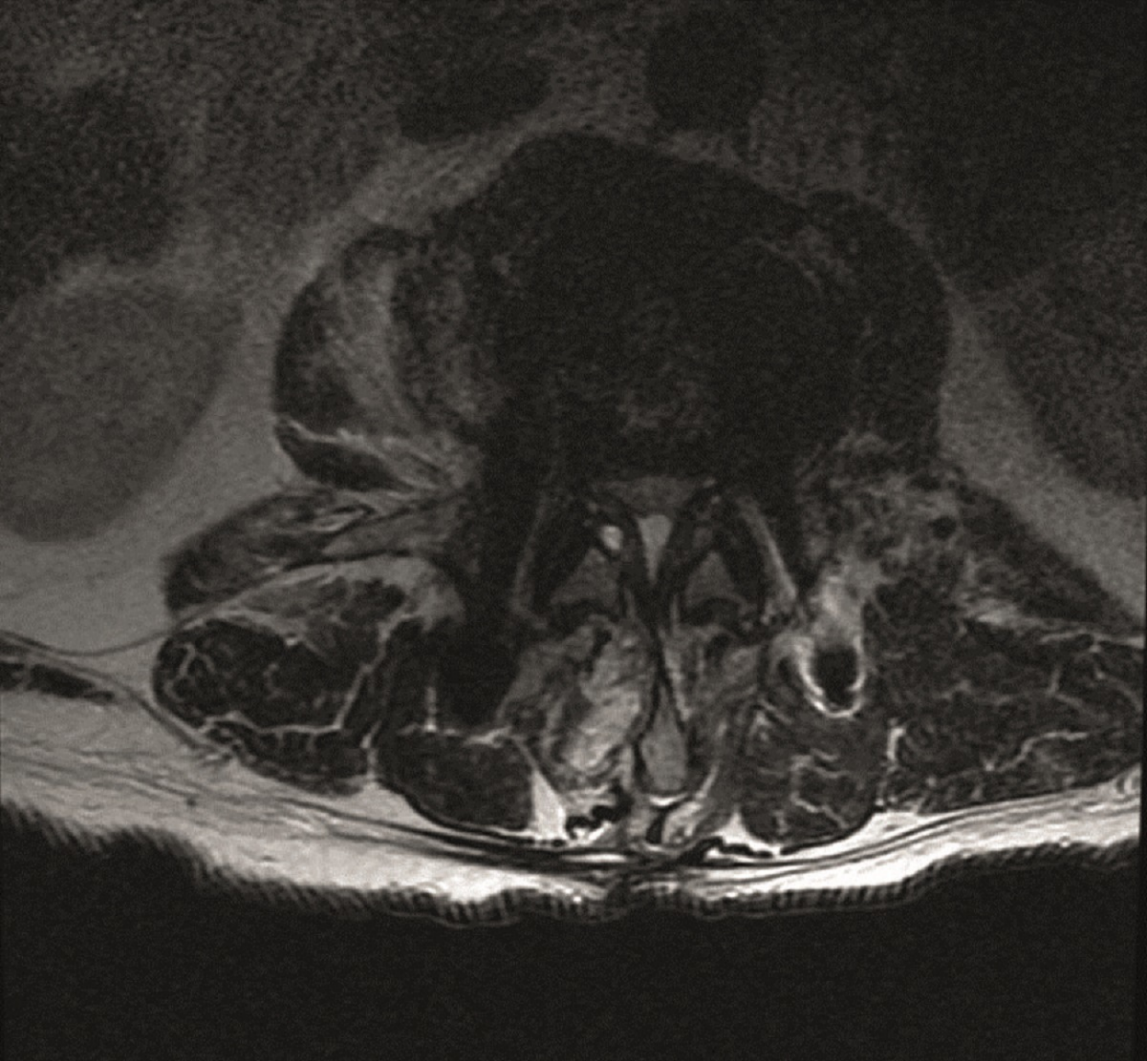
Axial T2 Pre-operative MRI Severe spinal canal stenosis at L2-L3 in association with a disc bulge and ligamentum flavum thickening and a small cyst on the right side.

**Figure 2:**
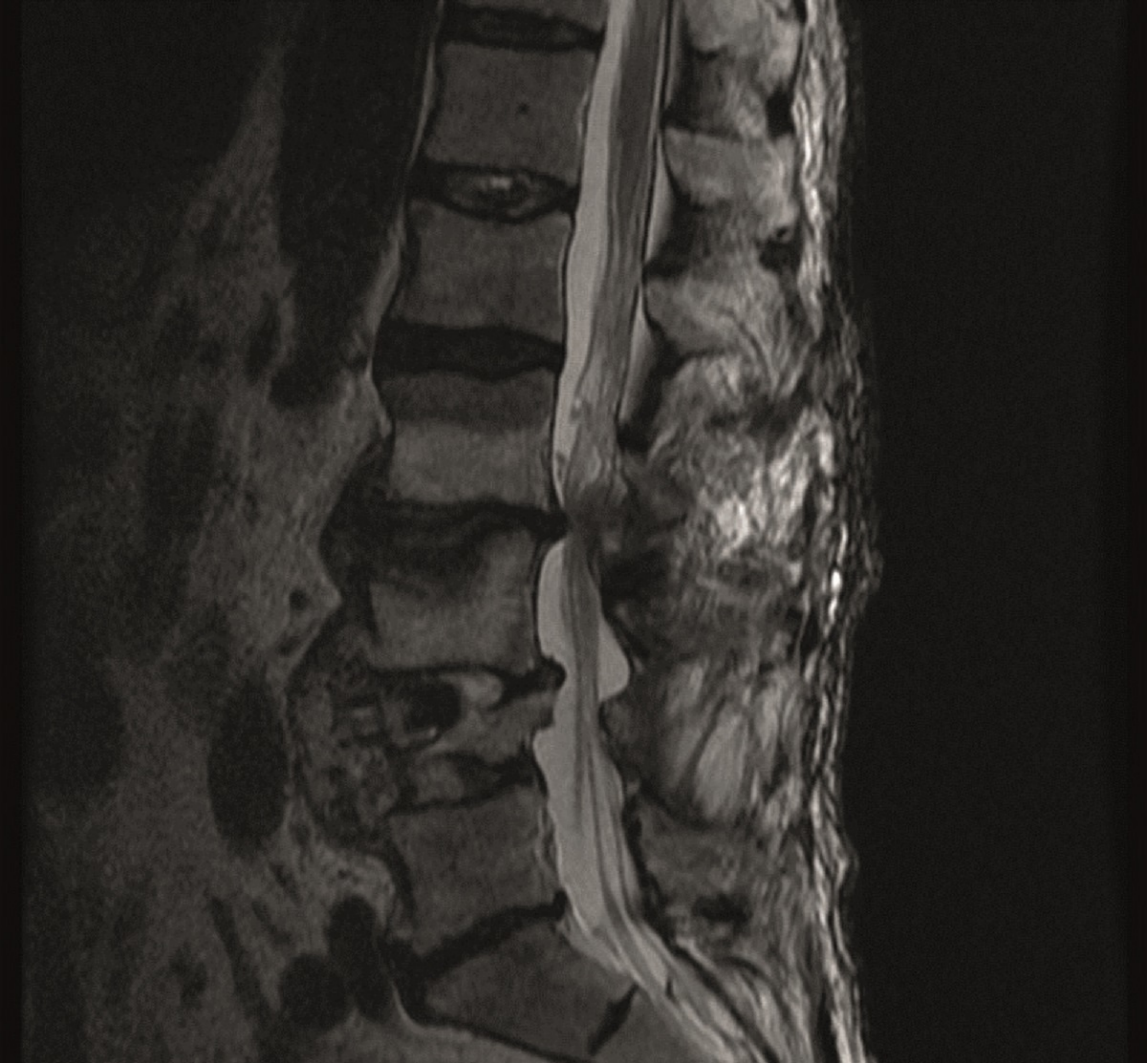
Sagittal T2 Pre-operative MRI

A 76-year-old male with a history of an L4 fracture with concomitant stenosis from L3-5 and instability underwent an L3-5 decompression and instrumented fusion with a laterally approached cage placement in December 2012. He recovered well for about one year, and then returned to the clinic with back pain, bilateral lower extremity heaviness, pain across his thighs, and difficulty ambulating long distances. Imaging revealed severe L2-3 stenosis, consistent with his symptoms of neurogenic claudication depicted in the axial and sagittal magnetic resonance imaging (MRI) in Figures 1-2.

**Figure 3:**
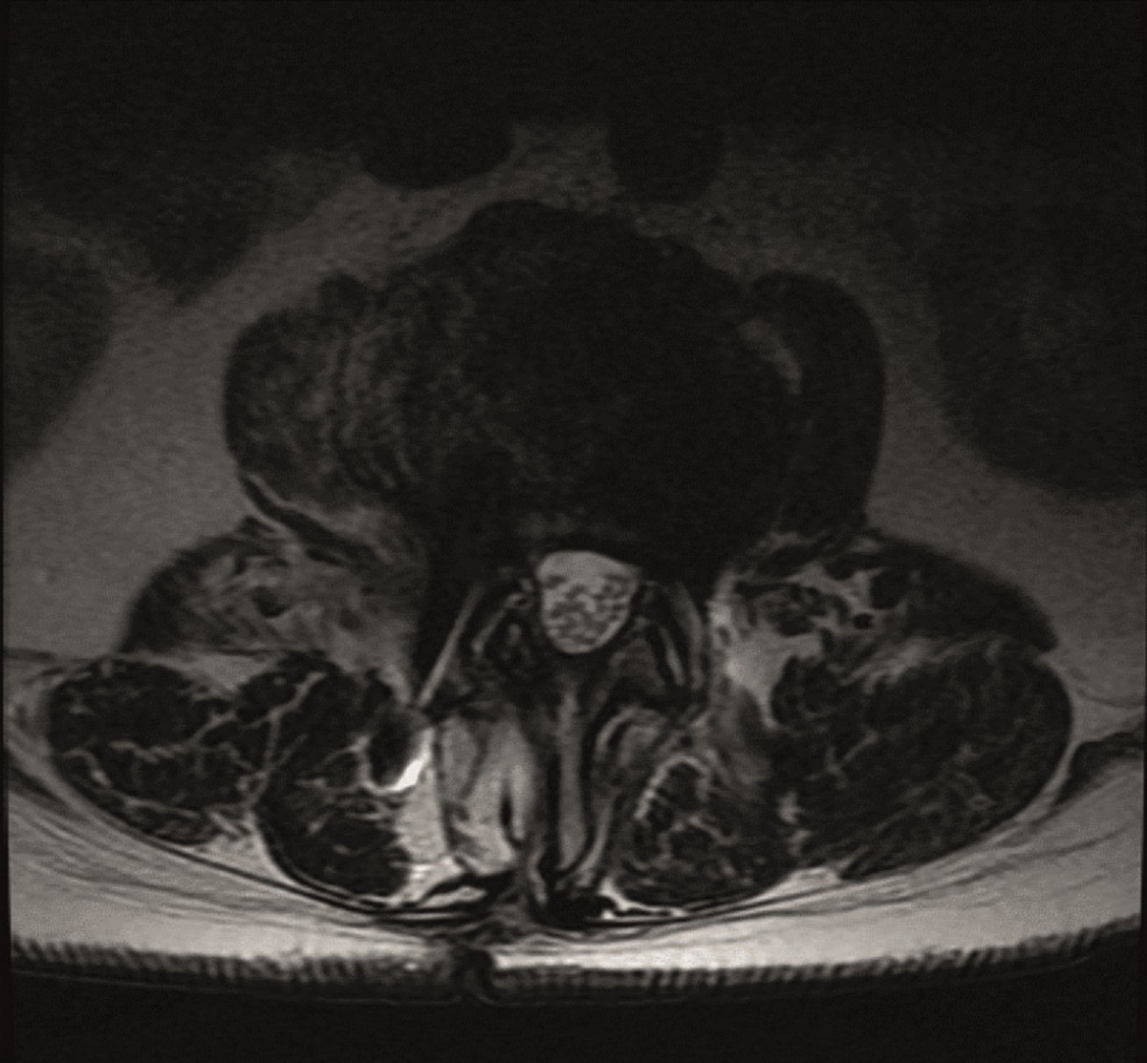
Axial T2 Post-operative MRI Interval marked widening of the spinal canal at L2-L3, with removal of the ligamentum flavum and the associated cyst.

**Figure 4:**
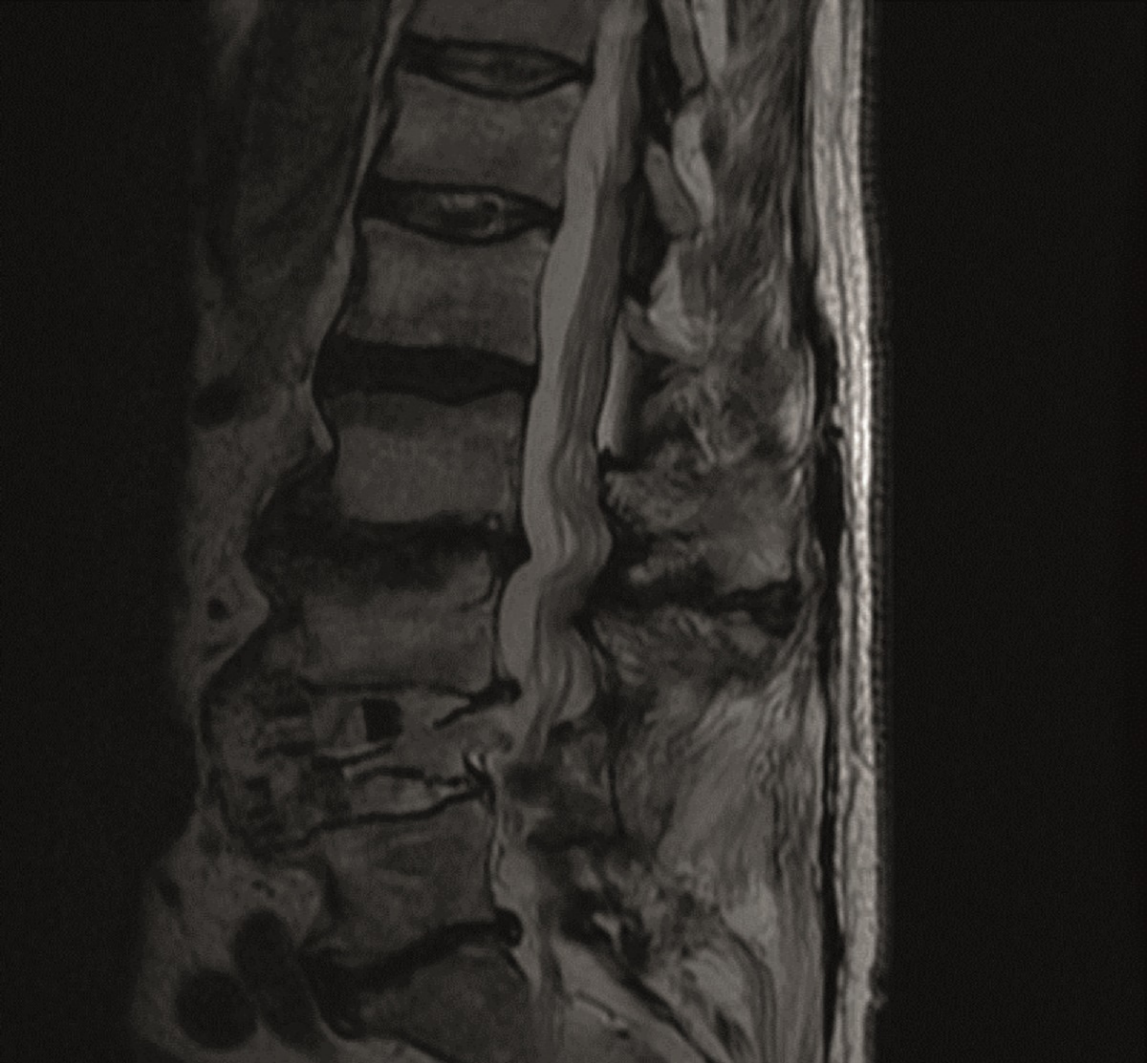
Sagittal T2 Post-operative MRI

He underwent a right-sided ULBD. He recovered well and was able to resume hunting. He underwent an MRI in October 2014 for unrelated reasons, and showed a well decompressed L2-3 bilaterally from the approach shown in the MRI in Figures 3-4.

### Technique

The ULBD is most easily performed at L2-3 and L3-4, but it can be considered at other levels as well, depending on the anatomy of the patient. The patient is placed in prone position on a Wilson frame. Once a localization X-ray has been performed, a midline incision is performed followed by a standard periosteal dissection of the para-spinal muscles unilaterally. An intraoperative X-ray will confirm the level. At this point the operative microscope or the SynaptiveBrightMatter^TM^Servo System (Synaptive Medical, Toronto, Canada) is brought in to complete the procedure. The sequencing of the procedure directs the remainder of the operation.

### Sequence 1

**Figure 5:**
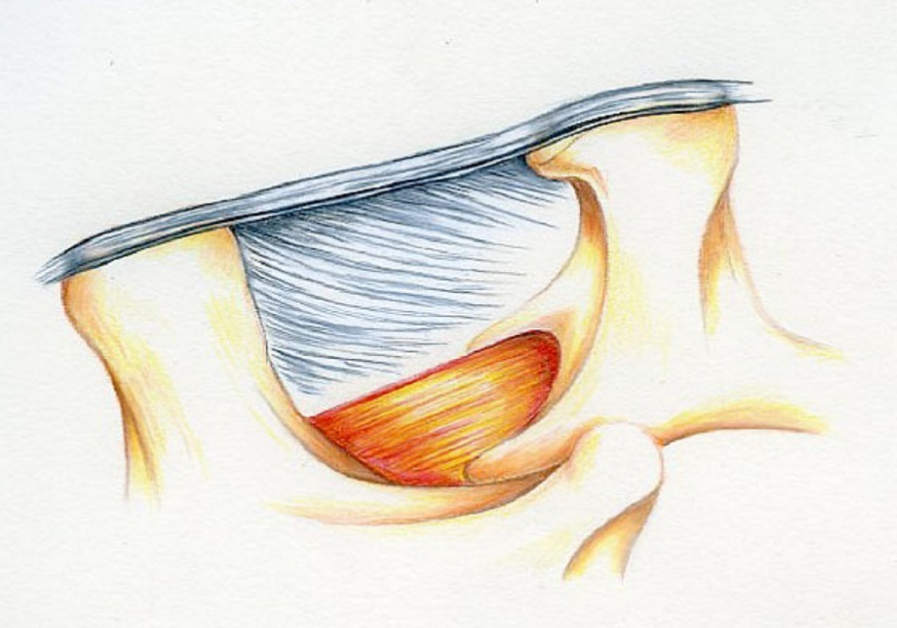
Pre-operative Illustration of L3-4 Laminar Space

We will assume this to be an L3-4 level for ease of presentation. An undisturbed depiction is shown in Figure 5. The goal of sequence 1 is to free up the ligamentum flavum from the inferior lamina of L3 on both sides.

**Figure 6:**
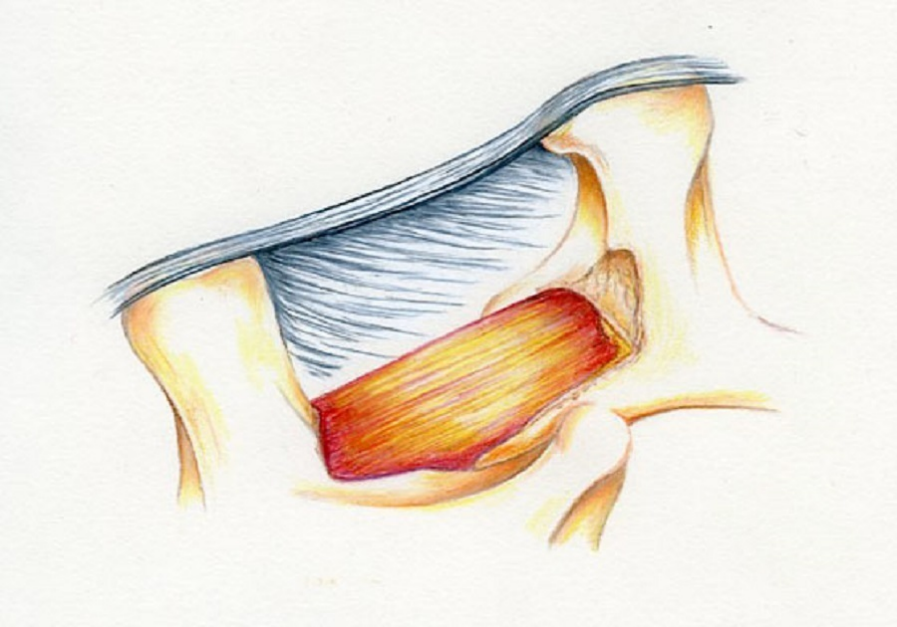
Illustration of Completed Sequence 1

A laminotomy is performed on the side of the incision. It is in the shape of an arch and the arch is extended anterior to the spinous process in the midline, and cephalad almost to the top of the ligamentum flavum. The flavum is completely released from the anterior lamina L3 with an angled curet, as illustrated in Figure 6.

The curet is moved only cephalad and caudad and no sweeping motions are made;  a severe dural tear can be caused by a sweeping motion from a curet.

The next step in Sequence 1 is to release the flavum on the contralateral side. A mark is made on the tip of the spinous process to indicate that about a third of it will be removed. A burr is used to remove this third of the tip of the spinous process and then the side of the spinous process is drilled down to the existing laminotomy. Progressively, more spinous process is removed as the dural sac is approached. By the time you connect the spinous process cut with the laminotomy you are almost level with the contralateral inferior lamina of L3. The table is rotated slightly away from the surgeon. The flavum is easily released with a Penfield 4 to the anterior border of the inferior lamina of L3. You can now directly visualize the plane between the flavum and inferior lamina of L3. The Penfield is swept superiorly to finish releasing the contralateral flavum from L3. Do not remove the flavum at this point. You can use a diamond or matchstick burr to widen the contralateral L3 if needed. This completes Sequence 1.

### Sequence 2

**Figure 7:**
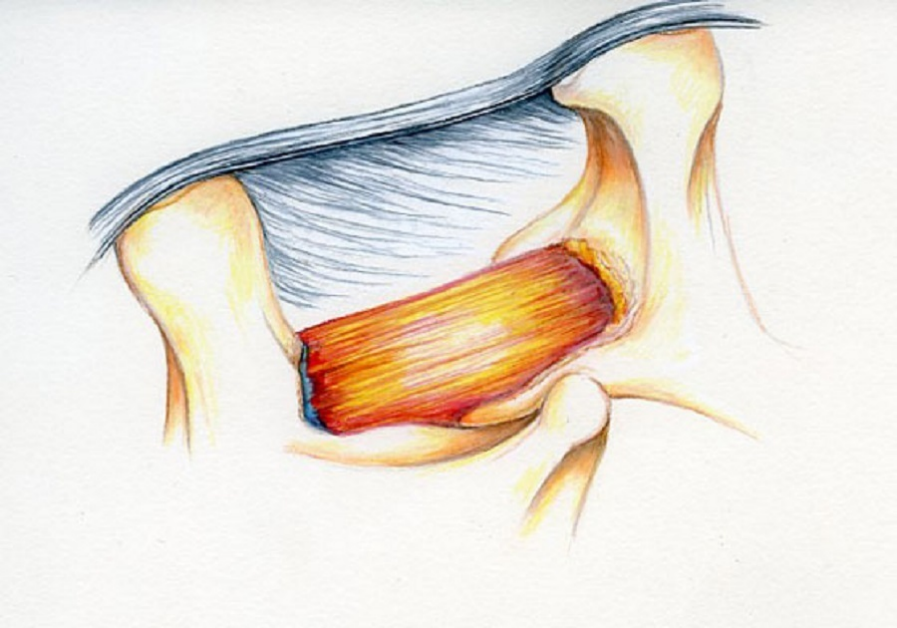
Illustration of Mid Sequence 2

The aim of Sequence 2 is to expose the lamina of L4 bilaterally and remove about 4 mm of lamina out to both L4 pedicles. A straight curet is used perpendicular to the floor to release the flavum from the superior edge on the ipsilateral side. A Kerrison punch is used to remove about 5 mm of lamina distally and towards the midline and partly towards the ipsilateral pedicle, as depicted in Figure 7.

**Figure 8:**
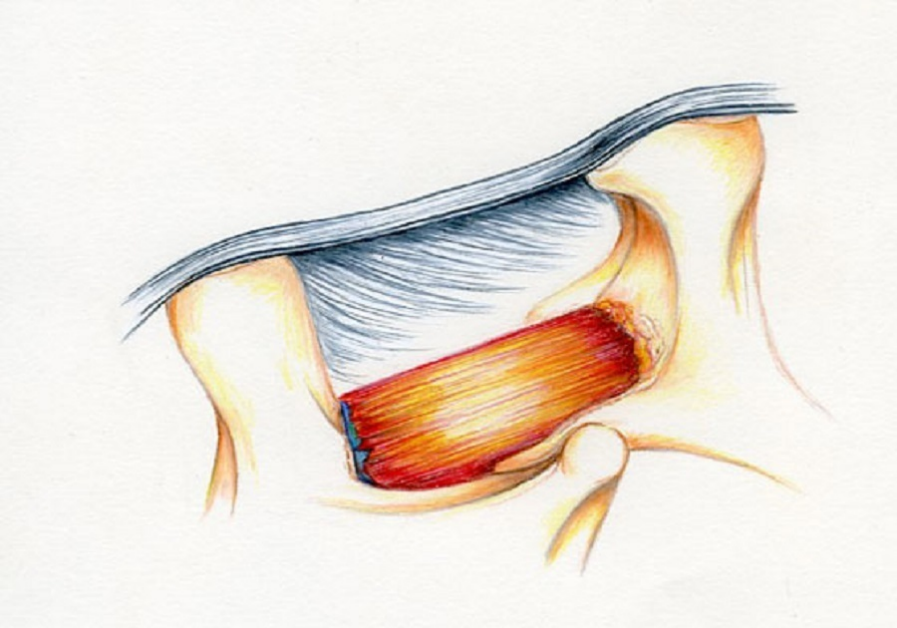
Illustration of Completed Sequence 2

Once the thickness of the lamina is determined, the midline “keel” or superior base of the L4 spinous process is removed with the burr parallel to the floor. This “keel” is flattened to achieve good visualization of the contralateral side. This is now connected with the tip of the spinous process with progressively more bone removed towards the contralateral side. The laminotomy is now enlarged towards the contralateral L4 pedicle, which is first palpated with a probe. You can visualize this pedicle and the contralateral L4 nerve root clearly. This is the contralateral completion of Sequence 2 and is elegantly illustrated in Figure 8.

### “The money move”

The bottom part of the recess of L4 has been removed out to the pedicle of L4. The table is now angled further away from the surgeon and the microscope is angled across the dural sac. A 3-mm Kerrison rongeur is slotted next to pedicle and angled cephalad about 30 degrees. The Kerrison is partially rotated towards the dural sac and the lateral recess is cracked and removed. The flavum attached to this remnant of the lateral recess will come out with the fragment and a significant amount of decompression is accomplished with this move. The next Kerrison move is over the top of the L4 pedicle and more lateral to the dural sac. An additional amount of flavum is removed and then the remaining flavum is removed from the shoulder of the L4 nerve root. This completes the contralateral “money move.”

**Figure 9:**
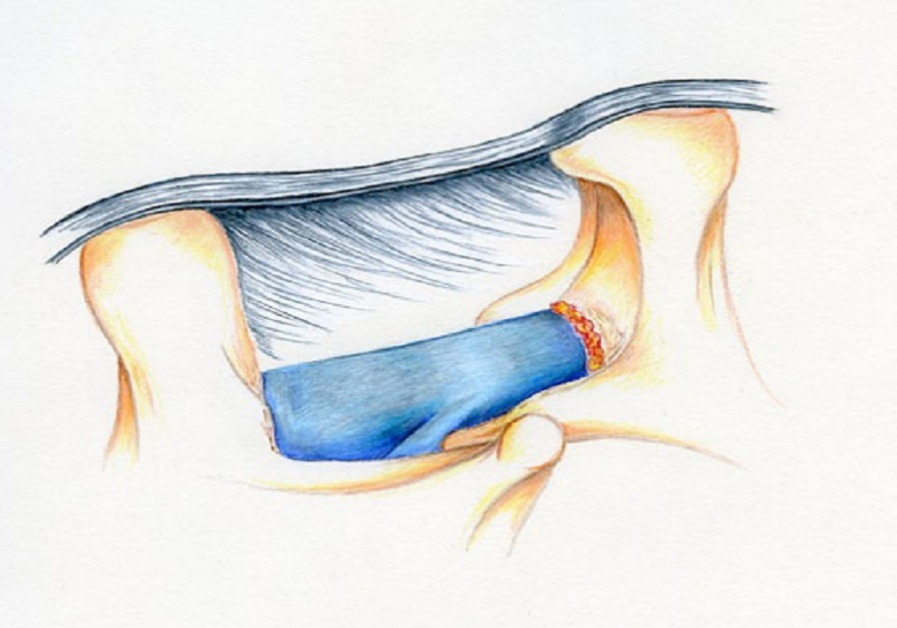
Illustration of Completed ULBD

The final phase of the procedure is to complete the money move on the ipsilateral side. Two thirds of the superior lamina of L4 has already been exposed and partially removed. The remaining recess is removed with a Kerrison at its origin from the pedicle and the attached flavum is removed from the shoulder of the L4 nerve. Again, the next Kerrison move is superior and lateral to the L4 pedicle and the rest of the flavum is removed from the shoulder of the L4 nerve. This completes the procedure, and the decompression is shown in its final form in Figure 9.

### Final comments

The result is essentially a midline laminectomy with the contralateral half of the spinous process and lamina completely intact. The chance of a dural tear is less on the contralateral side since you are biting away from the dural sac. It is important to work in a distal to proximal direction and to stay lateral to the dural sac. It is safer to remove the flavum from as anterior a point as possible. By starting the contralateral laminotomy at the tip of the spinous process you are able to visualize the edge of the dural sac and nerve root directly. It is important to leave the contralateral flavum in place to protect the dural sac during the initial dissection. We prefer to create a clean plane down to the anterior aspect of the contralateral inferior lamina. If there is a narrow canal at L4-5 then a ULBD may be preferable, but we think the benefit is marginal in a wide canal. L3-4 is occasionally wide enough for bilateral laminotomies to be preferable. We have occasionally removed large central discs with this approach, as the contralateral disc can be approached with a careful exposure. Caution is advised when the L3 lamina is encountered anteriorly to the spinous process; a fracture can be created at the base of the spinous process, which negates the potential benefits of the procedure.

## Discussion

Microsurgical ULBD is a decompressive approach to the lumbar spine used in selected cases where we want to preserve midline structure and the contralateral musculature. We believe that a systemized and detailed knowledge of the approach to this procedure will significantly improve clinical outcomes. Standard open laminectomy involves wide muscle retraction and extensive removal of the posterior spinal structures, which can sometimes lead to instability and the need for additional spinal fusion. Several studies have demonstrated the advantages of ULBD over standard open laminectomy for lumbar stenosis.

Mobbs et al. prospectively performed a 1:1 randomized clinical trial with an overall total of 79 patients enrolled, comparing ULBD to a standard open laminectomy. They concluded that ULBD is as effective as open decompression in improving function (Oswestry Disability Index (ODI) score). However, the ULBD procedure had additional benefits in terms of shorter length of stay (LOS) in the hospital, less time to mobilization, and less use of opioids for postoperative pain [1]. Yaman et al. retrospectively reviewed the data of 40 patients with lumbar spinal stenosis who underwent surgical treatment by comparing standard laminectomy with ULBD. As found by Mobbs et al., this study concluded that ULBD is an effective operation method with no instability effect and an increase in postoperative patient comfort [5]. In addition, Ho et al. evaluated the biomechanical stability of porcine lumbar spines after standard open laminectomy and unilateral as well as bilateral laminectomy, using a standardized motion tracking system. The open laminectomy group showed more instability than the unilateral and bilateral laminotomy groups [4].

Furthermore, Kakiuchi et al. retrospectively reviewed 48 patients who underwent lumbar open-door laminoplasty from 1993 to 2001, using a spinous process-splitting approach without disrupting the attachment sites of the multifidus muscle. Based on the ODI (p = 0.03), they demonstrated that the integrity of the spinous process after posterior decompression of the lumbar spine is a major traget for maintaining a positive surgical outcome [6].

In contrast, Thome et al. concluded that unilateral laminotomy is associated with a higher perioperative complication rate than bilateral laminotomy, including incidental durotomy, radicular deficits, and epidural hematoma [7]. We respectfully disagree as this is not our experience. We believe this is because we obtain better visualization of the contralateral side through our methodical approach.

It is suggested that the main disadvantage of this approach is that the surgeon has inferior vision to the contralateral site, the nerve root and the lateral gutter. However, in our experience the contrary is true. We think that you can attain excellent decompression of the contralateral side. We determined that the most important aspect is to make sure that the superior spinous process is removed, which allows direct visualization. We are even able to do a bilateral discectomy if necessary with this one-sided approach.

## Conclusions

A modified ULBD with partial removal of the ispsilateral half of the spinous process is superior to bilateral laminotomies at levels with a narrow canal. It has been demonstrated in the literature that preserving the spinous process is generally superior to the standard open laminectomy in terms of stability, level of invasiveness, and overall higher patient satisfaction.
